# The evaluation of Pat-Pat related injuries in the western black sea region of Turkey

**DOI:** 10.1186/1757-7241-19-40

**Published:** 2011-06-23

**Authors:** Sami Karapolat, Ayhan Saritas, Hayati Kandis, Mehmet Cikman, Suat Gezer, Ismet Ozaydin, Abdulkadir Iskender, Cagatay Calikoglu, Davut Baltaci, Mustafa Uslu, Banu Karapolat, Talha Dumlu

**Affiliations:** 1 Department of Thoracic Surgery, Duzce University School of Medicine, Duzce, Turkey; 2 Department of Emergency Medicine, Duzce University School of Medicine, Duzce, Turkey; 3 Department of General Surgery, Duzce University School of Medicine, Duzce, Turkey; 4 Department of Anesthesiology and Reanimation, Duzce University School of Medicine, Duzce, Turkey; 5 Department of Neurosurgery, Duzce University School of Medicine, Duzce, Turkey; 6 Department of Family Medicine, Duzce University School of Medicine, Duzce, Turkey; 7 Department of Orthopedics and Traumatology, Duzce University School of Medicine, Duzce, Turkey; 8 Department of Pulmonary Diseases, Duzce University School of Medicine, Duzce, Turkey

**Keywords:** Agriculture; Accidents, Traffic; Multiple Trauma; Emergency Service, Hospital; Emergency Treatment; Thoracic Surgery

## Abstract

**Background:**

Accidents caused by motorized vehicle in the agricultural sector are frequently observed. In Turkey; accidents arising from motorized vehicles, named Pat-Pat, which are used by farmers in the Western Black Sea region is not unusual.

**Methods:**

One hundred five patients who were brought into the Emergency Department of Duzce University, Medical Faculty Hospital between September 2009 and August 2010 due to Pat-Pat related accidents were evaluated.

**Results:**

The cases consisted of 73 (69.5%) males and 32 (30.5%) females, ranging from 2 to 73 years of age. In the 10-39 age group, a total of 63 (60.0%) cases were determined. The months when the greatest rate of cases applied to the hospital consisted of July, August, September and the season is summer. The cases were exposed to trauma in roads in 54 (51.4%), and 51 (48.6%) occurred in agricultural area without roads. Eighty seven (82.9%) cases were injured due to the overturning of vehicle. The patients were brought to the hospital using a private vehicle in 54 (51.4%) of the cases and in 51 (48.6%) cases, 112 ambulance system was used. The cases were determined to apply to the hospital most frequently between 6 pm-12 am. The injuries frequently consisted of head-neck and spine traumas, thorax traumas and upper extremity traumas. In 55 (52.4%) cases, open wound-laceration was determined. Seventy five (71.4%) cases were treated in the Emergency Department, and 28 (26.7%) were hospitalized. Three (2.9%) cases were deceased.

**Conclusions:**

Serious injuries can occur in Pat-Pat related accidents, and careful systematic physical examination should be conducted. In order to prevent these accidents, education of farm operators and engineering studies on the mechanics and safety of these vehicles should be taken and legal regulations should be created.

## Introduction

In Turkey, motorized vehicle related accidents are an important factor for morbidity and mortality, just like the rest of the world, and they are an important part of health requests [1]. Today, accidents due to motorized land vehicles that are used worldwide in the agricultural sector which includes production of vegetative products, improvement of quality and efficiency, conservation under proper conditions, handling, assessment and marketing of those products, make up a significant proportion of all motorized vehicle accidents due to properties of these vehicles and their different uses, security deficiencies, national control and licensing deficiencies, and their use by unlicensed or child agricultural workers [2]. These vehicles are designed to be used in ruined and hilly terrains in rural areas by agricultural and forestry sectors [3]. In the Western Black Sea region, due to mountainous terrain, stabilized fluctuating roads, in areas where tractors can not enter, motorized vehicles, named Pat-Pat, are frequently used by farmers, thus accidents are frequently encountered.

**Figure 1:**
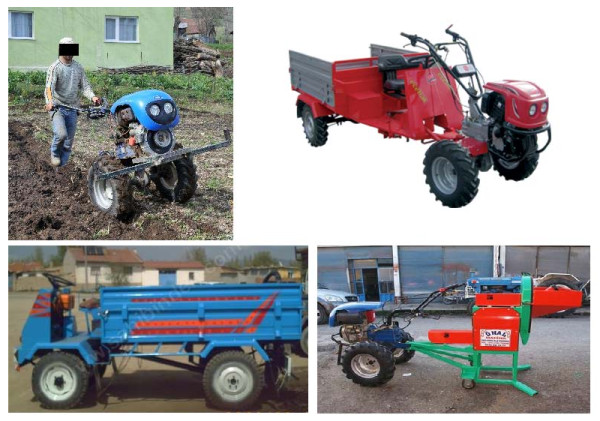
**Images of Pat-Pat and equipments**.

Pat-Pat farm vehicle structurally looks like an all-terrain vehicle (ATV), however it is functionally similar to farm tractors. It is well known in some regions of Turkey such as Duzce, Kocaeli and Sakarya, and named as "Pat-Pat". This name originates from the sound made by this machine. This vehicle has two separate structures; the main part is an engine and the other one is a trailer. Also, the engine part can be connected to other similar agricultural equipments (Figure 1). Pat-Pat's have five gears; four forward and one reverse and have a 12 horse power engine. This vehicle which weighs 300-350 kilograms on average and has models with steering wheels or handlebars can carry loads weighing more than one ton or 10-15 people. The recommended maximum speed on flat roads is 40 km/hr [4].

In agricultural areas, using appropriate equipments and special attachments Pat-Pat's can be used for in the expulsion of fields, planting, spraying activities, removing water from the soil, carrying additional load and even for transportation. Due to the lower price, lower fuel consumption, ease of use, its properties enabling it to be utilized in all weather conditions and it's ability to climb even the most steepest slopes, this vehicle practical, useful and increasing work-power. However, it had not become safer to use because of balance problems and lack of user protective properties such as roll-over protective structures.

In this study; we aimed to study the epidemiological and demographic characteristics of accidents caused by Pat-Pat's, present resulting health complaints, and investigating how to reduce the incidence and severity of these injuries.

## Materials and methods

Computer and hospital records from 105 patients who had applied to Duzce University Medical Faculty Hospital, Emergency Department between September 1, 2009 and August 31, 2010 due to motorized accidents with Pat-Pat's were examined retrospectively. The cases were assessed based on gender, age, monthly and seasonal cumulative distribution, regions where accidents took place and type of accident, time and method of transportation to hospital, localization of trauma, resulting pathologies, duration of observation in the Emergency Department, rate of discharge and hospital stay, and mortality rates arising during the early period.

In this study, for statistical analysis, "SPSS for Windows 11.5" software package was used. In the analysis of categorical variables, Chi-Square test (and/or Fisher's exact test) and for the comparison of means of two independent groups for numerical variables, Student t-test was preferred. The results are summarized as mean ± SD and p value less than 0.05 was considered as statistically significant.

The present study was carried out in accordance with The Declaration of Helsinki, and approved by the regional ethic committee.

## Results

Among 549 motorized vehicle accident patients that were admitted to the Emergency Department of our hospital between September 1, 2009 - August 31, 2010, 105 (19.1%) of them caused by Pat-Pat's. This study included 73 (69.5%) males and 32 (30.5%) females, ranging from 2 to 73 years in age. The mean age of males was determined to be 36.0 ± 16.6, and for females it was determined to be 32.2 ± 18.6. There was no significant difference between the two genders based on average age (p > 0.05). When age group distribution of cases exposed to trauma was examined the greatest frequency was found to be in the 10-39 years of age group with 63 (60.0%) cases (p < 0.0001) (Table 1).

**Table 1:** Distribution of the patients based on age groups

Age	n	%
**1-9**	4	3,8

**10-19**	19	18,1

**20-29**	24	22,9

**30-39**	20	19,0

**40-49**	12	11,4

**50-59**	15	14,3

**60-69**	7	6,7

**70-79**	4	3,8

The most frequent applications were discovered to be made during the months of July, August and September (n = 13, 12.4%, n = 37, 35.2%, and n = 15, 14.3%, respectively) (p < 0.0001). Based on seasonal applications, the greatest number of applications took place during summer (n = 56, 53.3%) (p < 0.0001). When the remaining seasons were examined, there were 35 (33.3%) cases during fall, 12 (11.4%) cases in spring and only two (1.9%) cases in winter.

Fifty four (51.4%) cases were exposed to trauma in roads and 51 (48.6%) in agricultural area without roads (p > 0.05). The injuries were due to vehicle being overturned in 87 (82.9%) cases, collision of Pat-Pat with another vehicle in 15 (14.3%) cases, and falling off the Pat-Pat in three (2.8%) cases (p < 0.0001).

Fifty four (51.4%) of the cases were brought into the hospital using private vehicles, whereas 51 (48.6%) were brought in using the 112 ambulance system (p > 0.05). The cases most frequently applied to the Emergency Department between the hours of 6 pm-12 am (n = 40, 38.1%). When the remaining times of admission were evaluated, it was observed that 38 (36.2%) cases were admitted between 12 pm-6 pm, 23 (21.9%) cases were admitted between 6 am-12 pm, and four (3.8%) were admitted between 12 am-6 am (p < 0.0001).

**Figure 2:**
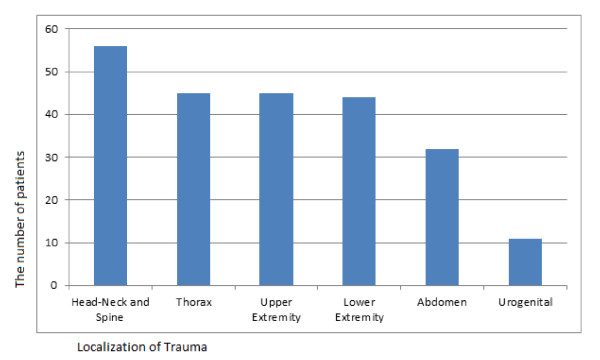
**Localization of trauma of the patients**.

**Figure 3:**
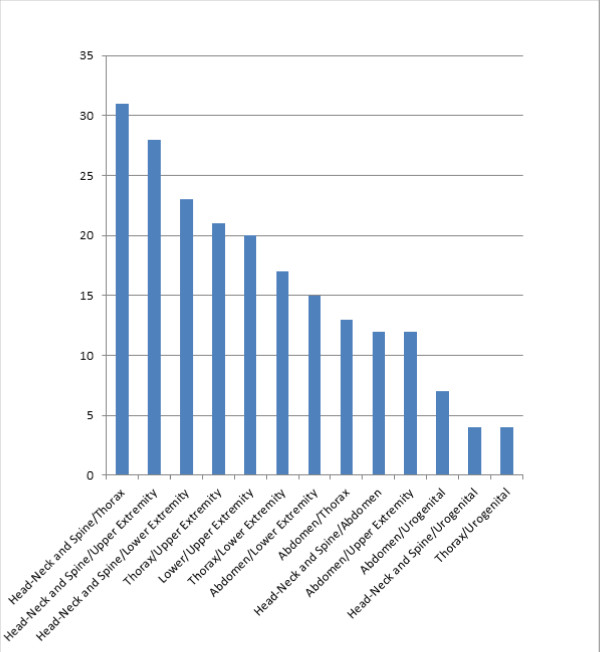
**Distribution of traumas related to more than one system**.

When resulting traumas were classified based on anatomical localization, most frequently, head-neck and spine trauma (n = 56, 53.3%) was observed, followed by thorax trauma (n = 45, 42.9%) and upper extremity trauma (n = 45, 42.9%) (Figure 2) (p < 0.0001). Frequently, head-neck and spine trauma was observed with thorax traumas (n = 31, 29.5%), and it was also observed to be accompanied with upper extremity traumas (n = 28, 26.7%). Traumas related to many systems were observed in only two (1.9%) cases (Figure 3). The open wound-laceration in 55 (52.4%), extremity fracture-dislocation in 34 (32.4%), internal injuries related to chest-abdomen-pelvis in 11 (10.5%), and intracranial lesions in six (5.7%) cases were observed (p < 0.0001).

After being treated in the Emergency Department, 75 (71.4%) of the cases were discharged. The average follow-up period of patients in the Emergency Department was 6.1 ± 2 hours (1-12 hours). Twenty eight (26.7%) of the cases were hospitalized. Most frequently, the patients were admitted to the Orthopedics Clinic (n = 12, 42.9%). The hospitalized cases were followed for at least 1 and at most for 82 days (mean: 13.2 ± 16.1 days). A total of three cases (2.9%); two from the Emergency Department and one from the reanimation unit were deceased.

## Discussion

In general, when agricultural injuries are examined, tractor-related injuries emerge as the most frequently seen accidents, which are more serious and have a higher rate of death [2,5,6]. In our study, approximately ^1^/_5 _of all motorized vehicle accidents were determined to be caused by Pat-Pat's. We believe this high rate to be due to Pat-Pat's being preferred for use in work in agricultural areas, and in addition to this, they are used for transportation and shipping purposed in roads. The deficiencies with regards to safety contribute to this high rate.

Based on the studies conducted, the rate of males who sustained traumas during tractor accidents, were observed to vary between 72.5-77% [6,7]. Similarly, in our study also, this rate for males was determined to be 69.5%. In addition, although we encountered Pat-Pat related injuries in all age groups, we observed a higher frequency of patients in the 2, 3, and 4 decade age groups. These results can be explained by the high density of young population living in our region and the presence of male-dominated society from a sociological perspective. With the region's main income source being agriculture, almost all young individuals play an active economical role, earning an income, not letting the elderly work, thus increasing the probability of accidents related to agricultural occupations and explaining the high accumulation observed in the above decades.

When a general assessment is made, it is discovered that the number of cases is relatively small during winter, then with spring this number increases and peaks during summer months and stays relatively high until the end of fall. This result is highly affected by agricultural workers and the population, whose income depends on agriculture, starting to work on farms during these months. We believe that another reason for traumas frequently seen during the months of July, August and September, is the hazelnut cultivation which requires hazelnut garden and tree maintenance, and collection of hazelnuts during these months.

We observed that majority of Pat-Pat accidents have occurred in roads and the rest occurred in agricultural area without roads. This result is an indication that Pat-Pat's are used in highway transportation as much as they are used in the agricultural sector which they were originally designed and marketed for. We believe that the increase in the traffic volume in many rural roads of our county in the recent years and large speed difference between motor vehicles and Pat-Pat's, have lead to the increase in Pat-Pat related accidents in roads.

Some studies show that, tractor overturn has been the most commonly reported cause of occupational injury and death in the agricultural related injury [2,8,9]. In Pat-Pat's whose speed limit can be doubled by modifications, balance problems can easily occur on certain surfaces, due to an insignificant mistake made by the driver or driving through a sharp curve in the road, as a result loss of control of the vehicle and overturning can cause serious traumas. A majority of the accidents in this study have occurred to Pat-Pat's overturning. As a result of rolling, the driver and passengers who are not wearing any protective equipment such as helmets or seat belts are thrown onto the road or under the vehicle. In our study, three patients who were deceased were thrown under the vehicle.

Carlson et al. have determined that most of the tractor-related injuries (82%) occurred between the hours of 6:00 am and 5:59 pm [6]. In our study, throughout the day, the number of patients applying starts increasing in the afternoon and peak at evening hours. As a reason for this, we believe that accidents occur more during evening hours because the collected produce is transported for storage, the workers need to be brought to their homes and increased highway traffic.

In this study, the most affected localizations of trauma consisted of head-neck and spine, thorax and upper extremities. Among these patients, thoracic injury victims had a different type of management within the routine procedures of our clinic. Among the 45 thorax trauma cases, 21 (46.7%) had single or multiple rib fracture, 14 (31.1%) had pulmonary contusion, 11 (24.4%) had clavicle fracture, nine (20.0%) had pneumothorax, eight (17.8%) had subcutaneous emphysema, five (11.1%) had hemopneumothorax, four (8.9%) had hemothorax, four (8.9%) had intraparanchimal hematoma, two (4.4%) had flail chest, two (4.4%) had pulmonary laceration, one (2.2%) had traumatic asphyxia, one (2.2%) had right intermediate bronchus rupture, and one (2.2%) had left diaphragm rupture. Among these patients, in case of those who had non-complicated rib fractures of three or less and who did not have additional pathologies, the length of the follow up period in the Emergency Department was kept relatively longer; at the 6. hour, control chest roentgenograms were taken and if no problems developed, they were discharged after proper medical treatment. Among the 28 (26.7%) patients who were treated after being admitted to the hospital, one or more kind of thorax trauma varieties listed above was present in 24 of them. Throughout the study, of the four patients who were admitted to the Chest Surgery Clinic, two had subcutaneous amphysema + multiple rib fractures + hemopneumothorax, one had pneumothorax + pulmonary contusion and one patient had left diaphragm rupture. Although, in general, the cause of death in 25% of fatal trauma cases consisted of thorax trauma, in this study, it has been proven that in pathologies related to chest surgery, less invasive surgical methods, such as tube thoracostomy, can be life-saving and sufficient for treatment in a majority of cases [10]. Among these cases, we conducted primary repair of the diaphragm of the patient who had left diaphragm rupture via thoracotomy, the patient who had right intermediate bronchus rupture had bronchoplasty via thoracotomy, and in one the patients who had pulmonary laceration, we carried out the primary repair of the lacerated area via thoracotomy. In other cases, a total of 21 tube thoracostomies was conducted and a different surgical treatment was not necessary.

In ATV related accidents, in 45-67% of cases, in addition to the head-neck and extremity combinations, majority of injuries involved more than one organ system [3,11-13]. We determined injuries related to more than one organ system in 66.7% of patients. Due to this high ratio, we believe that the physical examination of these patients should be detailed and inclusive of all systems; even in the case of normal findings, before being discharged, repeat systemic physical examination should be conducted in order to eliminate possible pathologies that may occur later on.

In more than half of our patients, open wound-lacerations and in approximately ^1^/_3 _of them, extremity fracture-dislocation were determined. This situation explains the reason why most patients are admitted to the Orthopedics clinic. This result suggests that, accidents related to Pat-Pat's may have at least as serious consequences as other tractor-related accidents [14-19].

We treated approximately ¾ of the patients in the Emergency Department. After necessary treatments, these patients were kept under observation slightly longer than routine Emergency Department patients, those who were stabilized and did not have any problems were discharged. The hospitalization rate in our study is 26.7%. The social hospitalization indication has increased this rate due to most of the population living in our region residing in rural areas away from the city center, which do not have medical facilities.

These injuries can be significantly damaging and frequently, they can be prevented. The passing laws allowing the regulation of sale, maintenance, traffic record and use of these vehicles, and training drivers about the vehicle's properties, emphasizing the features that they need to pay close attention, seem to be the most important precautions. Since Pat-Pat related accidents frequently occur in roads, the enforcement of laws that are passed, and their effective control, would be more possible. In addition, attendance of farmers to educational programs for usage of Pat-Pat's by local agriculture societies and traffic center workers, will add to the solution. Although any investigation regarding alcohol level of the patients during at the time of accident in the present study, topic regarding avoidance of alcohol use while driving a vehicle should be emphasized at educational program for farmers, based on knowledge of alcohol intake which usually increases markedly frequency of traffic accidents. As the medical community, we believe that a multidisciplinary approach by government agencies, rural politicians, civil society organizations, written-visual media and commercial organizations would contribute greatly towards this goal.

This study has a few limitations such as the small number of patients, the scope of the study covering only a period of one year, the lack of operator-passenger differentiation, results of breathalyzer of the drivers, and the lack of long-term follow up. We believe that additional studies where greater numbers of cases are examined through the contribution of other large trauma hospitals in our region, their demographic and injury characteristics are assessed, Pat-Pat's age, speed and technical properties are investigated, where, as a result, specific risk factors are determined, will increase the value of our remarkable findings.

## Conclusion

In the Pat-Pat associated accidents, physicians should keep in mind that the injuries may be as serious as those caused by other bigger motorized vehicles, and a comprehensive physical examination should be conducted. Additionally, the observation period should be longer than the routine practice, and physicians should not refrain from admitting those to the hospital. In fact, traffic accidents are a public health problem, thus, instead of considering just treatment services as being sufficient, preventative measures should also be considered as a necessary part of the bigger picture. Thus, in order to prevent Pat-Pat accidents, it is crucial to create legal regulations, conduct further engineering studies regarding the mechanics, design and safety of the vehicles, license the vehicles, periodically inspect them, and train users with educational programs on this subject. The development of a nationally organized program where all these injury prevention strategies are combined, which target farm operators and workers, will be one of the most important steps in this issue.

## Competing interests

The authors declare that they have no competing interests.

## Authors' contributions

SK, AS, HK, MC and SG participated in the design of the study and coordination, data analysis, literature search, and writing/revision of manuscript. IO, AI and CC supervised the study and performed the statistical analysis. DB conceived of the study, and participated in its coordination. MU has given assistance to analyzing the data. BK and TD have contributed in collecting the data and literature search. All authors read and approved the final manuscript.
